# Clinical and Imaging Findings of True Hemifacial Hyperplasia

**DOI:** 10.1155/2013/152528

**Published:** 2013-09-19

**Authors:** Bansari A. Bhuta, Archana Yadav, Rajiv S. Desai, Shivani P. Bansal, Vipul V. Chemburkar, Prashant V. Dev

**Affiliations:** ^1^Department of Oral Pathology, Nair Hospital Dental College, Dr. A. L. Nair Road, Mumbai, Maharashtra 400 008, India; ^2^Department of Radiology, Topiwala National Medical College & B. Y. L. Nair Hospital, Mumbai 400008, India

## Abstract

Congenital hemifacial hyperplasia is a rare developmental disorder of unknown etiology, characterized by a marked unilateral facial asymmetry. It involves the hard (bones and teeth) and soft tissues of the face. We report an interesting case of true hemifacial hyperplasia in a 25-year-old male highlighting the clinical and computed tomography imaging findings.

## 1. Introduction

Congenital hemihyperplasia is a rare developmental disorder characterized by unilateral overgrowth of one or more body parts resulting in marked asymmetry. This phenomenon was first described by Meckel [1] in 1822 and first reported by Kottmeier and Wagner [2] in 1839. Rowe [3] in 1962 classified hemihypertrophy into (1) complex hemihypertrophy, involving the entire half of the body, (2) simple hemihypertrophy affecting one or both limbs, and (3) hemifacial hypertrophy. Depending on involvement of soft tissues, teeth, and bones, he further classified hemifacial hypertrophy into (a) true hemifacial hypertrophy and (b) partial hemifacial hypertrophy. True hemifacial hypertrophy exhibits unilateral enlargement of all tissues, teeth, bones, and soft tissues, characterized by viscerocranial enlargement, bounded by frontal bone superiorly (sparing the eye), inferior border of the mandible inferiorly, midline medially, and ear including the pinna laterally. In partial hemifacial hypertrophy not all structures are enlarged to the same degree or limited to one structure. We prefer to use the term congenital hemifacial hyperplasia although it is usually referred to as hemifacial hypertrophy. The term hyperplasia is more precise histologically, as all tissues show an increase in the number of cells rather than an increase in cell size [4]. The asymmetry usually remains constant with the end of adolescence, and as the skeletal maturation occurs the condition stabilizes thereafter. 

We report an interesting case of true hemifacial hyperplasia (THFH) in a 25-year-old male, discussing clinical features, imaging findings, and differential diagnosis in detail to supplement the current literature.

## 2. Case Report

**Figure 1:**
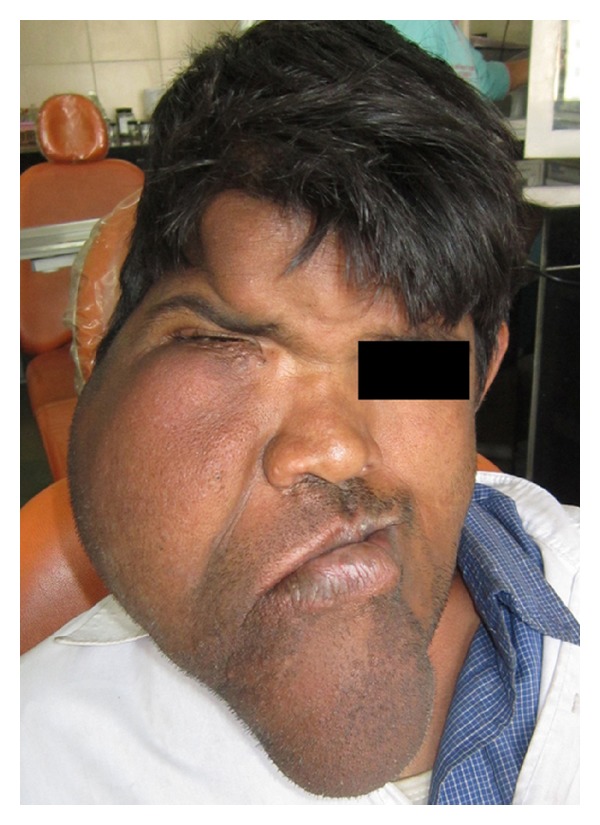
Unilateral enlargement of right side of the face. Note the asymmetry of the forehead, cheeks, nose, lips, chin, and the closed eye.

A 25-year-old male reported to our department for opinion regarding a massive, asymptomatic enlargement of the right half of the face since childhood (Figure 1). The asymmetry had increased with age and ceased to grow after 18 years of age. Family history was unavailable since he was an orphan. Medical examination did not reveal any other health issues. The patient had deferred treatment until now due to the lack of financial resources. Extraoral examination revealed a massive and diffuse enlargement of the right side of the face. The enlargement extended from the midline to the preauricular region, superiorly to the frontal bone and inferiorly to the inferior border of mandible. The nose and chin were deviated towards the left side of the face, with the facial midline describing a gentle arc from nasion to gnathion instead of the usual vertical straight line. The right corner of the mouth was drooped and lips were larger on the right side. The skin of the involved right side of the face was coarser than the unaffected left side. The right pinna was larger than that of the left side. There was monstrous enlargement of the soft tissue over the zygoma, the infraorbital region and the symphyseal region. The enlarged soft tissue mass on the right side of the face caused closure of the right eye, compromising the patient's vision. There appeared to be an excessive increase in size of the right side of the mandible, maxilla, zygoma, and frontal bone, as compared to the contralateral side. On palpation a nontender, soft tissue swelling over the right parietal bone was noticed. No temporomandibular joint disorder or dysfunction was detected. No discrepancy in range of mandibular motion was noted.

**Figure 2:**
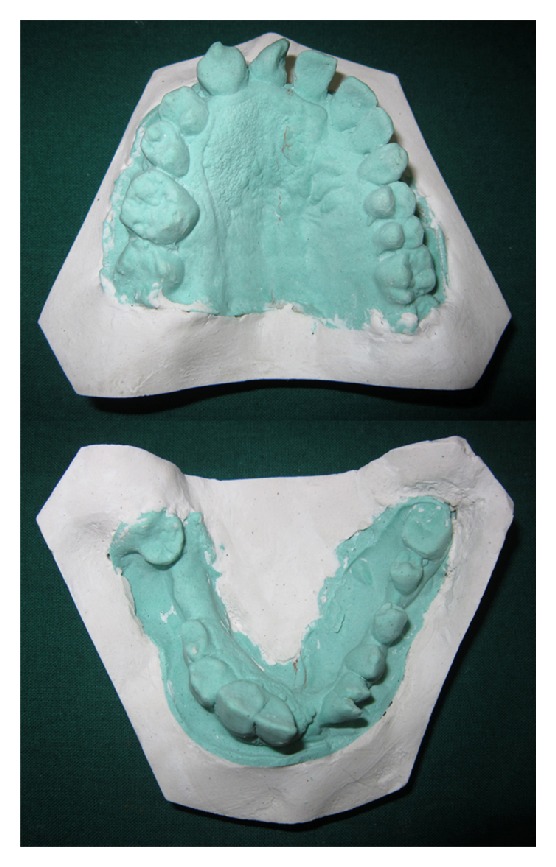
Occlusal view of the maxillary and mandibular dental casts showing macrodontia of the right side, midline shift to the left, along with granular surface of the right palatal mucosa.

**Figure 3:**
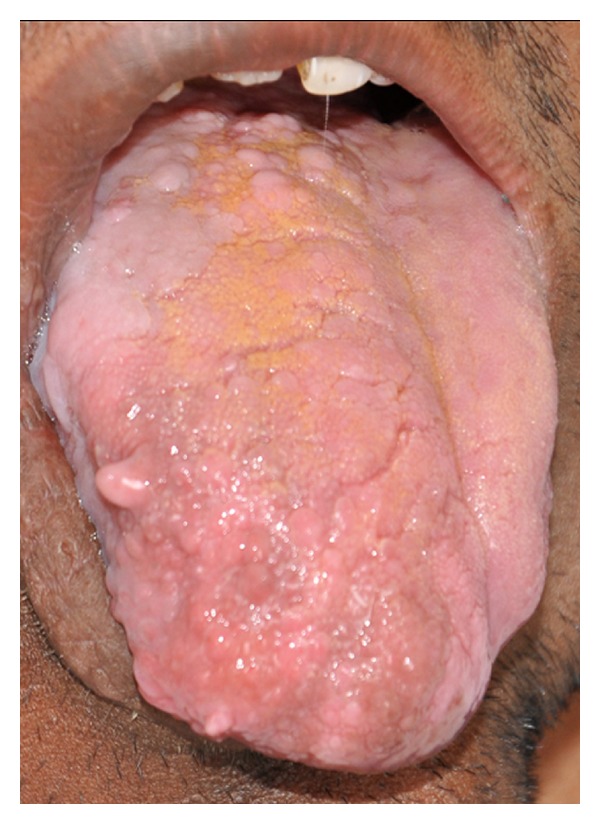
Intraoral photograph showing enlargement of right side of the tongue with enlarged papillae.

**Figure 4:**
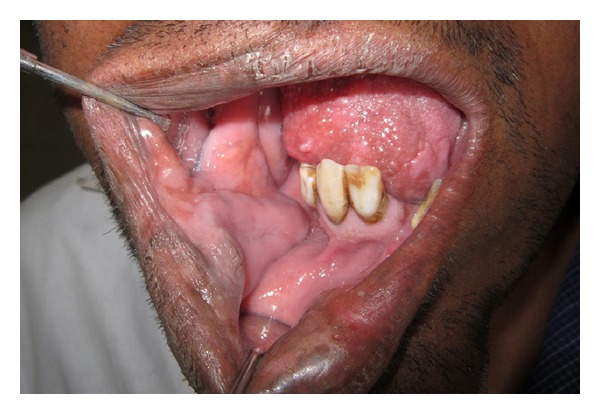
Intraoral photograph showing velvety buccal mucosa hanging in pendulous folds and granular gingival surface along with macrodontic permanent right lateral incisor and canine.

**Figure 5:**
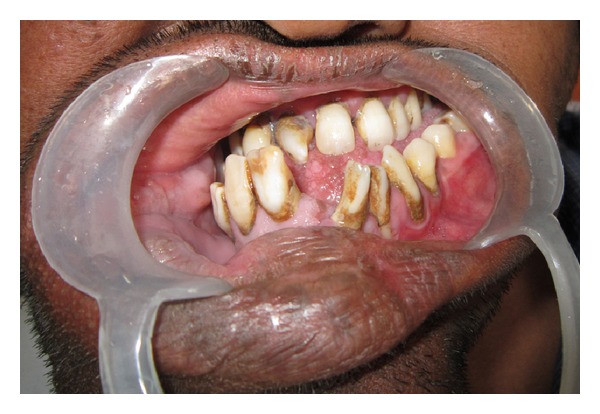
Intraoral photograph showing major variability between crown sizes of the teeth on the right and the left side with generalised crossbite relationship.

Intraoral examination disclosed an obvious alveolar enlargement of the right maxillary and mandibular quadrants as compared to the left quadrants (Figure 2). The surface of the gingival tissue and palate on the right side was granular in appearance. Right half of the tongue showed an obvious enlargement to the midline with polypoid excrescences representing enlargement of the fungiform papillae (Figure 3). The right buccal mucosa was thickened and hung in pendulous folds (Figure 4). A distinct tooth size discrepancy was observed between right and left side. Detailed coronal dimensions of the teeth were measured on the casts with vernier calipers, which revealed major variations in size between the teeth of the affected side compared to the uninvolved side. Right permanent maxillary canine, first premolar, second premolar, first molar, and second molar were larger cervicoincisally, mesiodistally, and labiolingually than those of the left side (Table 1). Similarly, right permanent mandibular lateral incisor, canine, first premolar, and second premolar were larger cervicoincisally, mesiodistally, and labiolingually than those of the left side (Table 2). The above mentioned right maxillary and mandibular teeth were considerably enlarged than their contralateral counterparts. The right permanent maxillary canine, first premolar, second premolar, first molar, and second molar demonstrated 224%, 100%, 63%, 110%, and 75% increase in volume, respectively, than their contralateral counterparts, while the right permanent mandibular lateral incisor, canine, first premolar, and second premolar demonstrated 337%, 150%, 116%, and 57.14% increase in volume, respectively, than their contralateral counterparts. The maxillary and mandibular midline was shifted to the left. A generalised crossbite was present due to a prognathic mandible (Figure 5). The occlusal plane on the right side was canted downwards.

**Figure 6:**
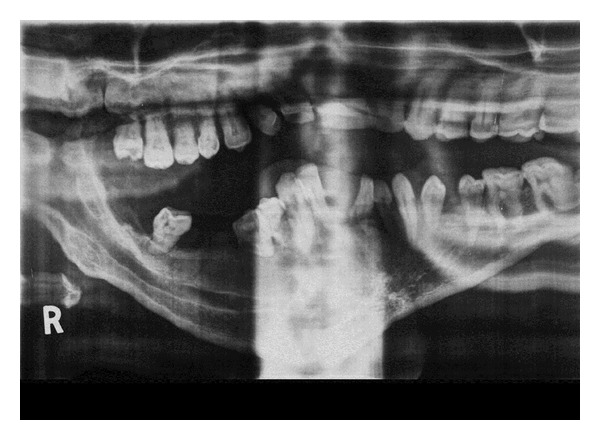
Panoramic radiograph showing widening of the right inferior alveolar canal.

**Figure 7:**
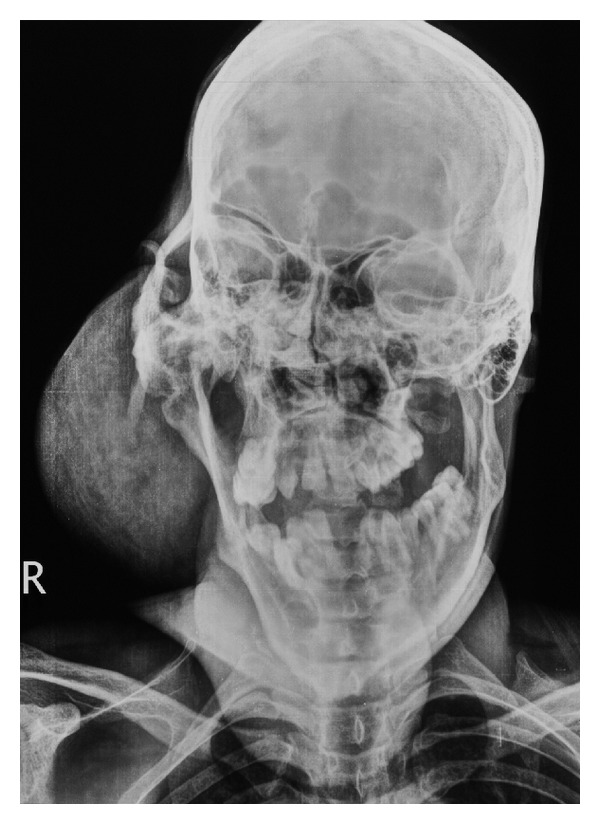
Posteroanterior view of the skull showing appreciable generalized bony and soft tissue enlargement of the right face.

The panoramic radiograph showed enlarged right body of the mandible with the widening of the right inferior alveolar canal (Figure 6). PA Skull showed enlargement of right half of mandible, maxilla, and zygoma (Figure 7). Soft tissue enlargement was seen on the right side of the face and also encircling the symphysis. 

**Figure 8:**
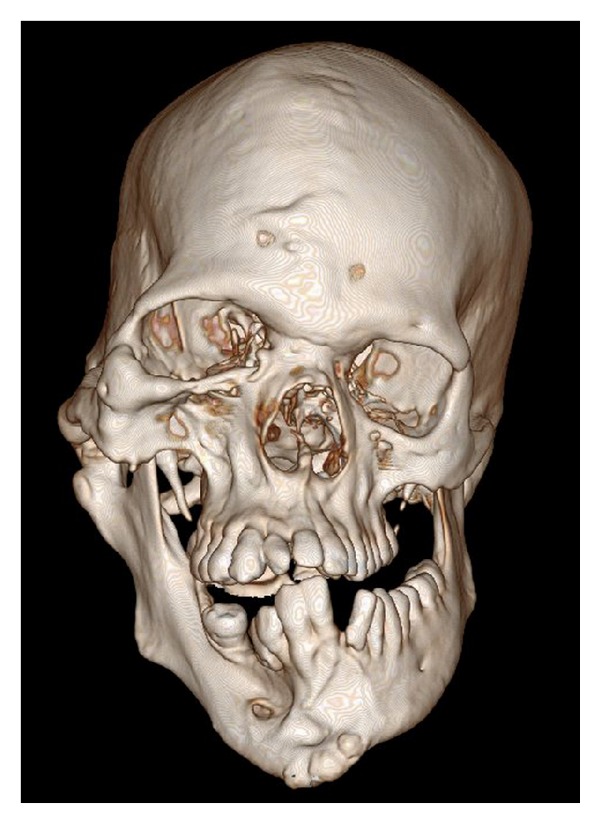
3D volume rendered CT scan showing enlarged mandible, maxilla, and zygoma, with enlarged right mental foramen and teeth of the right side.

**Figure 9:**
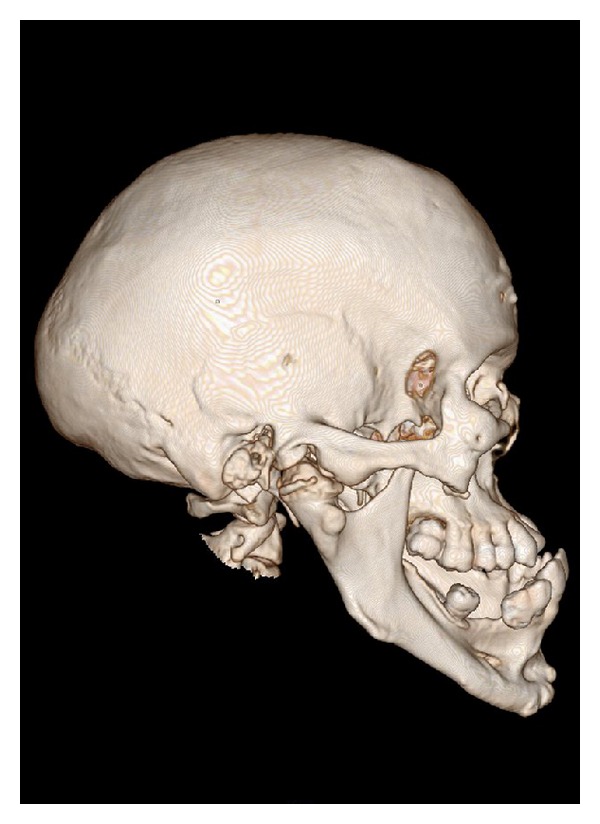
Lateral view of 3D volume rendered CT scan showing enlarged right condyle, coronoid process, mandibular body, and zygoma.

**Figure 10:**
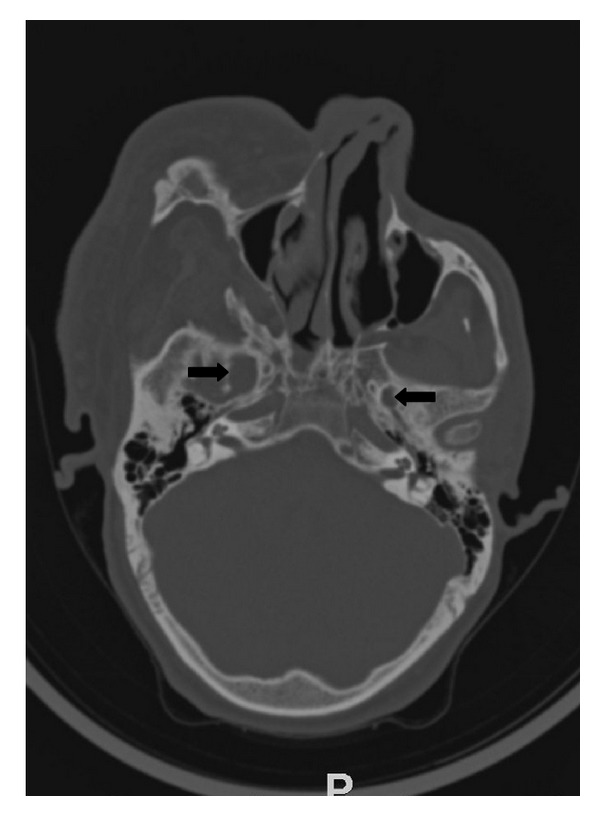
Axial CT scan with bone reconstructions showing widened foramen ovale (right side-arrow, left side-arrowhead).

**Figure 11:**
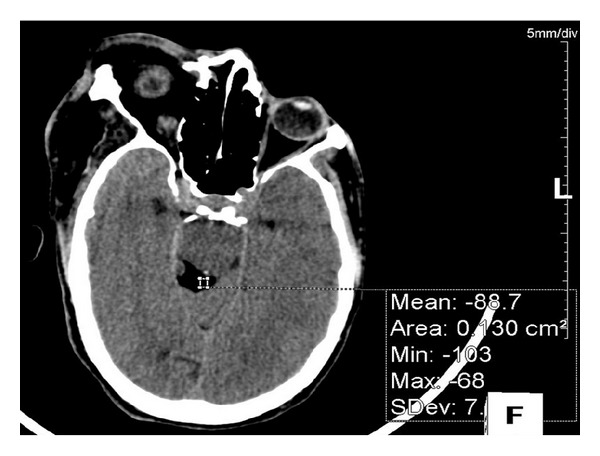
Axial CT scan showing a lipoma in the right quadrigeminal cistern.

**Figure 12:**
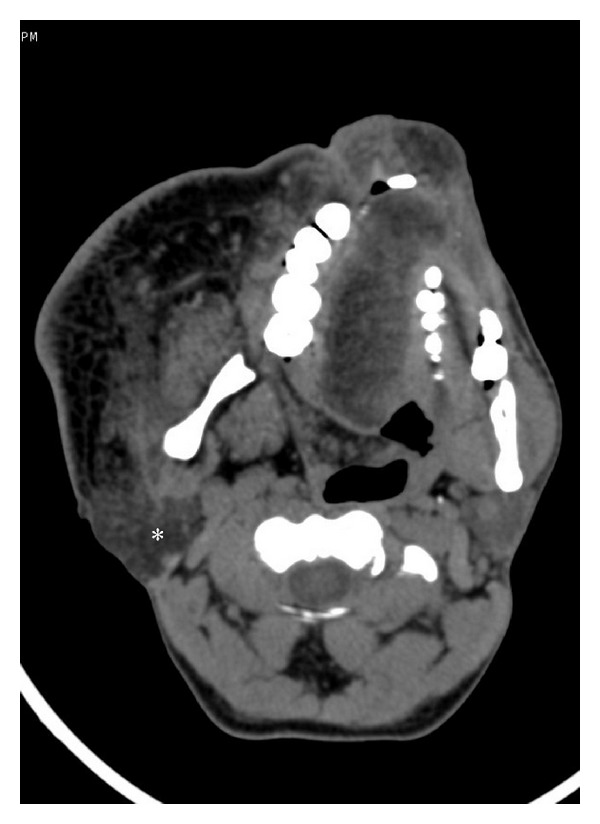
Axial CT scan showing lipomatous enlargement of the soft tissue on right side of face including buccal region, lips, tongue, soft palate, and right parotid gland (asterix).

**Figure 13:**
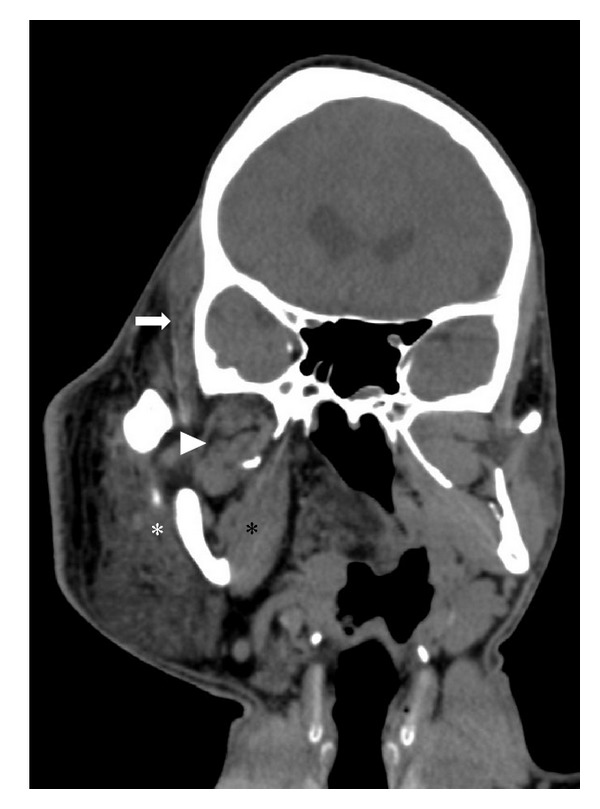
Coronal CT scan revealing hyperplasia of the right mandibular condyle, medial pterygoid (black asterisk), lateral pterygoid (arrowhead), masseter (white asterisk), and temporalis muscle (white arrow).

**Figure 14:**
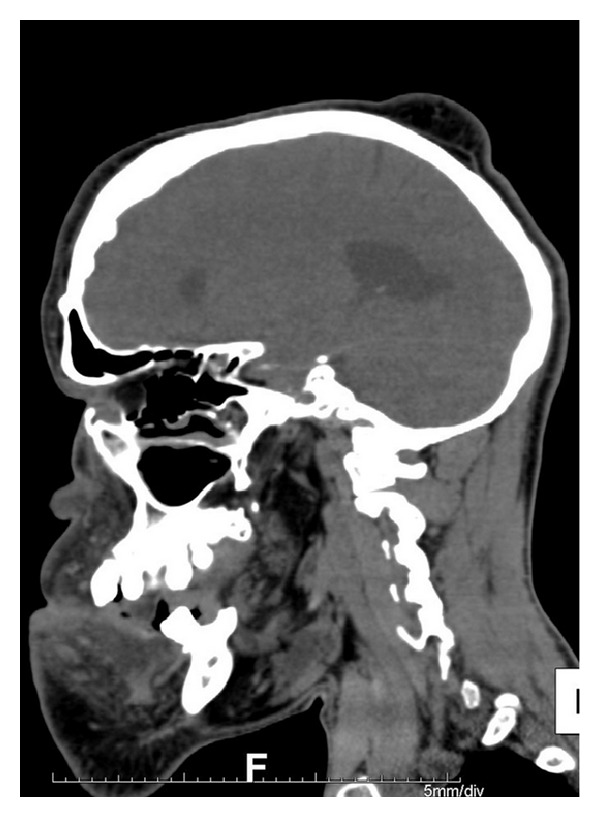
Sagital CT scan showing soft tissue swelling in right high parietal region.

Computed tomographic (CT) scan of face revealed enlargement of the bony structures, including right half of maxilla, mandible, condyle, zygoma, bony walls of external auditory canal, and pterygoid bone (Figures 8 and 9). There was bony overgrowth of the glenoid fossa with irregularity of the articular surface. The right condyle was found to be irregular and flattened; however, the temporomandibular joint space was maintained. The right external auditory canal appeared stenosed due to bony overgrowth. The right frontal and parietal bones were thinned out with irregularity of inner table. The right foramen ovale, spinosum, rotundum, mental and infraorbital foramen, vidian canal, and greater and lesser palatine canals were widened as compared to those of the left side (Figure 10). An intracranial lipoma in the quadrigeminal cistern of the right side was also seen (Figure 11). Bony orbit on right side was deformed; however, globe, intra-, and extra-orbital structures were normal. Deformation and deviation of the nasal bone and chin were seen towards the left side due to enlargement of overlying soft tissues. Prominent vessels and few nodular serpiginous areas were seen within right parotid gland which was enlarged with heterogenous appearance (Figure 12). The right submental region, submandibular region, parapharyngeal space, pterygopalatine fossa, soft palate, tongue, and floor of mouth were involved (Figure 12). All the muscles of mastication and the anterior belly of diagastric on the right side were enlarged with fatty infiltration (Figure 13). Soft tissues of the right half of the face were hypertrophied, which demonstrated predominantly fat HU (Hounsfield unit) value (Figures 12 and 13). A 5.4 × 4.7 cm sized soft tissue swelling was seen in right high parietal region with fat HU value suggestive of lipoma (Figure 14). 

Based on clinicoradiological findings, the diagnosis of THFH was established. Multiple reconstructive procedures were advised in view of correction of the massive facial deformity. The patient, however, refused to undertake the extensive surgeries, since the enlargement was asymptomatic.

## 3. Discussion

Subtle asymmetric variations of contralateral structures of the face and head are common occurrences and can be esthetically enhancing. However, marked unilateral overdevelopment of the hard and soft tissues of the head and face is a rare congenital malformation variously described as facial hemihypertrophy, partial or unilateral gigantism, and hemifacial hyperplasia [5]. 

Hemifacial hyperplasia (HFH) was first reported by Friedreich in 1863 [6]. The prevalence rate of HFH is 1 : 86000 live births [7]. It affects men more commonly than women with the right side of the face more commonly affected than left side as observed in the present case [3]. Whites are more commonly affected than blacks [8]. HFH may be associated with other conditions, such as acromegaly and pituitary gigantism, or with hypertrophy of other parts of the body [8]. 

The etiology is unknown, but the condition has been ascribed to vascular & lymphatic malformations, endocrine disorders, neurocutaneous lesions, and central nervous system lesions leading to altered neurotropic action, abnormal intrauterine environment, somatic mutations, mechanical influences, and congenital syphilis [8]. Gesell suggested that the condition may result from a deviation of the normal process of twinning [9]. Noe and Berman have reviewed that fusion of two eggs following fertilization leads to unequal regulative ability in two halves, and mitochondrial damage to an overripe egg leads to overregeneration [10]. Pollock and colleagues have proposed a new embryologic hypothesis of asymmetrical development of the neural fold and hyperplasia of the neural crest cells as the basis for this disorder [4]. According to Yoshimoto et al., the basic fibroblast growth factor (FGF) and its receptor may be selectively involved in the facial asymmetry, stimulating an osteoblast DNA synthesis of the affected side in a more pronounced manner than that of the unaffected side [11].

HFH is associated with a wide variety of abnormalities such as thickened skin and hair on the involved side, excessive secretion of sebaceous and sweat glands, and vascular and pigmentary defects of the affected side [12]. Different texture and colour variance of ipsilateral scalp hair have also been reported [4]. In addition skeletal abnormalities such as macrodactyly, polydactyly, syndactyly, ectrodactyly, scoliosis, tilting of pelvis, and clubfoot have also been described [13]. Central nervous system involvement in the form of cerebral enlargement, epilepsy, strabismus, and mental retardation in 15–20% of patients has been reported in the literature [14]. Ipsilateral pinna and pupil may be enlarged, but an increase in size of the inner ear or globe of the eye has not been reported [4]. Occurrence of small exostoses of the posterior auditory canal has also been reported [12]. Adrenal cortical carcinoma, nephroblastoma (Wilm's tumour), and hepatoblastoma can be occasionally associated with this disorder [15]. Genitourinary system disorders, such as hypospadias, cryptorchidism, and medullary sponge kidney, were also noted occasionally [13]. 

Soft tissue like lips, uvula, and tonsils may be involved, and frequently the tongue exhibits enlarged lingual papillae with unilateral enlargement and displacement towards the normal side [4]. The buccal mucosa may also be involved exhibiting velvety surface and hangs in soft pendulous folds as reported by Miles [16]. Our patient presented with prominent enlargement of the tongue, lingual papillae, soft palate, and buccal mucosa. The mass of the ipsilateral parotid gland and that of the muscles of mastication were increased in our patient as reported by many authors [4, 13]. Widening of the palate and enlargement of the alveolar bone of the affected side were noted in the present case as described in the literature [4]. According to Rowe, the size and shape of the tooth crown and root size, as well as rate of development, are usually abnormal if the teeth are affected [3]. Random increase in tooth size frequently affecting cuspids, followed by premolars, molars, and incisors has been reported [17, 18]. Usually enlargement does not exceed 50% of normal side. The size of the teeth crown was considerably enlarged on the affected side than on the nonasffected side in our patient. 

Hemifacial hyperplasia may occur as a part of crossed hemifacial hyperplasia in which facial asymmetry along with a coexistent enlargement of the opposite lower extremity is present [5]. Hemifacial lipomatosis may represent a possible subtype of partial hemifacial hyperplasia in which lipomatosis is a prominent feature [19]. Hemifacial myohyperplasia is also newly proposed and described as a separate entity in which there is predominantly hyperplasia of the facial musculature. In cases with hemifacial myohyperplasia, there is an enlargement of the muscle mass of the facial muscles with ipsilateral hypoplasia of the facial skeleton [20]. Our patient demonstrated both hyperplasia of the muscles of mastication and presence of lipomas along with involvement of bones and teeth. The enlargement of all tissues on the right side of the face and the absence of limb enlargement lead to the diagnosis of THFH.

While evaluating a patient with HFH, acquired causes of cranial asymmetry such as benign fibro-osseous lesion like fibrous dysplasia, Paget's disease, and dyschondroplasia (Ollier's disease), and malignant conditions like osteosarcoma and chondrosarcoma should be considered. Congenital hyperplasia shows overgrowth of bone and soft tissue with foraminal enlargement, while the other conditions do not show this feature [4]. There was enlargement of right foramen ovale, spinosum, rotundum, mental and infraorbital foramen, vidian, canal and greater and lesser palatine canals in our patient which distinguishes HFH from other entities. In addition, all these conditions have distinct features that differ from those of HFH radiographically and clinically. Occasionally, hemifacial atrophy (Parry-Romberg syndrome) can mimic hemifacial hyperplasia on the normal side; however, these patients present with atrophy later in life between 5–15 years of age. Sometimes HFH may resemble cystic hygroma, especially if the lymphatic anomaly is located inferior to the jaws and in the neck region. But HFH is a solid noncystic disease, whereas cystic hygroma is fluid filled and yields an aspirate which is high in fat content.

Other malformation syndromes and abnormalities such as neurofibromatosis, Proteus syndrome, Beckwith-Wiedemann syndrome, Schimmelpenning (epidermal nevus) syndrome, hyperpituitarism, Maffucci syndrome, Ollier's syndrome, Langer-Giedion syndrome, Klippel-Trenaunay-Weber syndrome, McCune-Albright syndrome, Russell Silver syndrome, triploid/diploid mixoploidy, multiple exostosis syndrome, and segmental odontomaxillary dysplasia may superficially resemble HFH. However, the unilateral distribution of dental anomalies and the concurrent unilateral tongue enlargement are the prominent features which make HFH an unique entity [3].

Our patient had the classical clinical features of THFH such as unilateral overgrowth of the orofacial soft tissues, tongue, teeth, and bones causing an obvious asymmetry of the right side of the face. CT scan of the present case demonstrated prominent facial asymmetry which was caused by the unilateral enlargement of both soft tissue structures and the underlying skeleton of the right side of the face, along with dental involvement, making this case unique in its presentation. 

An extensive search of the English literature revealed no formal reports of malignant degeneration in HFH [8]. Hemifacial hyperplasia is generally associated with good prognosis. Treatment is not indicated for HFH unless cosmetic considerations are involved. 

## Figures and Tables

**Table 1:** Comparison of crown sizes of the maxillary teeth.

Maxillary arch										
Dimension (mm)	Canine		1st premolar		2nd premolar		1st molar		2nd molar	
	L	R	L	R	L	R	L	R	L	R
Cervicoincisal	11	17	9	12	9	10	9	12	9	11
Mesiodistal	7	11	8	9	7	8	11	13	10	13
Labiolingual	6	8	6	8	7	9	9	12	10	11
Total surface volume (cu. mm)	462	1496	432	864	441	720	891	1872	900	1573
Enlarged by	224%		100%		63%		110%		75%	

L: left; R: right.

**Table 2:** Comparison of crown sizes of the mandibular teeth.

Mandibular arch								
Dimension (mm)	Lateral incisor		Canine		1st premolar		2nd premolar	
	L	R	L	R	L	R	L	R
Cervicoincisal	9	15	14	17	10	13	8	11
Mesiodistal	6	9	8	11	7	10	8	8
Labiolingual	4	7	6	9	6	7	7	8
Total surface volume (cu. mm)	216	945	672	1683	420	910	448	704
Enlarged by	337%		150%		116%		57.14%	

L: left; R: right.
